# BOP1 Knockdown Attenuates Neointimal Hyperplasia by Activating p53 and Inhibiting Nascent Protein Synthesis

**DOI:** 10.1155/2021/5986260

**Published:** 2021-01-16

**Authors:** Fangyuan Jia, Qi Wu, Zhiwei Wang, Min Zhang, Shun Yuan, Yanjia Che, Bowen Li, Zhipeng Hu, Xiaoping Hu

**Affiliations:** ^1^Department of Cardiovascular Surgery, Renmin Hospital of Wuhan University, 238# Jiefang Road, Wuhan, 430000 Hubei Province, China; ^2^Cardiovascular Surgery Laboratory, Renmin Hospital of Wuhan University, 9# Zhangzhidong Road, Wuhan, 430000 Hubei Province, China; ^3^Central Laboratory, Renmin Hospital of Wuhan University, 9# Zhangzhidong Road, Wuhan, 430000 Hubei Province, China

## Abstract

The rate of ribosome biogenesis plays a vital role in cell cycle progression and proliferation and is strongly connected with coronary restenosis and atherosclerosis. Blocking of proliferation 1 (BOP1) has been found as an evolutionarily conserved gene and a pivotal regulator of ribosome biogenesis and cell proliferation. However, little is known about its role in neointimal formation and its relationship with vascular smooth muscle cell (VSMC) proliferation and migration. The present study mainly explores the effect of BOP1 on VSMCs, the progression of neointimal hyperplasia, and the pathogenic mechanism. The expression of BOP1 was found to be significantly elevated during neointimal formation in human coronary samples and the rat balloon injury model. BOP1 knockdown inspires the nucleolus stress, which subsequently activates the p53-dependent stress response pathway, and inhibits the nascent protein synthesis, which subsequently inhibits the proliferation and migration of VSMCs. Knockdown ribosomal protein L11 (RPL11) by transfecting with siRNA or inhibiting p53 by pifithrin-*α* (PFT-*α*) partly reserved the biological effects induced by BOP1 knockdown. The present study revealed that BOP1 deletion attenuates VSMC proliferation and migration by activating the p53-dependent nucleolus stress response pathway and inhibits the synthesis of nascent proteins. BOP1 may become a novel biological target for neointimal hyperplasia.

## 1. Introduction

Coronary atherosclerosis and restenosis after percutaneous coronary intervention (PCI) is the main coronary artery disease (CAD) that seriously threatens human health. Neointimal hyperplasia is the common pathological change of CAD [1]. Even with a range of effective treatment, particularly drug-eluting stent implantation, the present state of treatment is not encouraging. In addition, recent evidence suggests that there are no significant differences in the prognosis between patients using drug-eluting stents and patients receiving bare-metal stents within a four-year observation period [2]. Coronary restenosis is still the major complication. Hence, there is an urgent need to identify a more efficient biological target.

The proliferation and migration of VSMCs play a pivotal role in neointimal hyperplasia, which are also mainstream therapeutic targets [3]. Accumulating evidence has indicated that the rate of ribosomal biogenesis is closely correlated with cell proliferation and migration. Ribosomes are mainly comprised of ribosomal protein (r-protein) and ribosomal RNA (rRNA) [4, 5]. The maturation of rRNA during ribosomal assembly mainly depends on the PES-BOP1-WDR12 complex (PeBoW-complex), which is thereby indispensable to ribosomal assembly and ribosomal biogenesis [6, 7].

BOP1 is a WD-repeat protein with five WD repeat motifs, is highly conserved in mammalian cells, and has been identified as the core modulator of the PeBoW complex [8]. Furthermore, BOP1 in charge of the biogenesis of the 28S and 5.8S ribosomal RNA (rRNA). The dominant negative mutant of BOP1 has been proven to inhibit cell proliferation by interfering with the multiple steps in pre-RNA processing [9, 10]. The repressing BOP1 function may alter the chromosome segregation or inhibit the cell migration ability via inhibiting RhoA and the actin stress fiber assembly [11], while overexpressing BOP1 increases the production of multipolar spindles in cells, which contributes to the progression of colorectal tumorigenesis [12]. The previous research conducted by the investigators revealed that BOP1 induces aortic media degeneration (AMD) in the process of aortic aneurysm/dissection (AA/AD) through accelerating p53-dependent VSMC apoptosis and oxidative stress [13]. Hence, it was assumed that BOP1 may be closely correlated to vascular homeostasis and play a vital role in neointimal hyperplasia.

As the site of ribosomal biogenesis, a variety of biological processes occur in the nucleolus, including the RNA polymerase-I (Pol-I) transcription of pre-RNA and the nuclear import and nucleolar accumulation of ribosomal r-proteins, and eventually, several events would induce the mature ribosomal subunits to be exported to the cytoplasm [14, 15]. In addition, the nucleolus can sense a variety of stress stimuli and trigger several response pathways to maintain cell homeostasis called nucleolar stress. This is critical for regulating cellular biological processes. Cellular stress stimuli inhibit the Pol-I or pre-rRNA modification, consequently releasing some r-proteins from the nucleolus to the nucleus, and thereby activating the p53-dependent or p53-independent stress response pathway. Importantly, r-proteins translocate from the nucleolus to the nucleoplasm and bind to murine double minute 2 (MDM2), thereby promoting p53 stabilization, which is known as the r-protein-MDM2-p53 stress response pathway. This has been widely demonstrated in the cellular response to nucleolar stress [16–20]. Among r-proteins, RPL11 is of great importance in the nucleolar stress response pathway. The central role of the RPL11/RPL5/5S-RNA ribosomal subcomplex (5S RNP) in the p53-dependent nucleolar stress signaling pathway has been reported by Sloan et al. [21], and this has been subsequently confirmed in various cell lines. Although both BOP1 and nucleolar stress have been shown to be closely correlated to ribosome synthesis, the correlation between BOP1 and 5S RNP in vascular smooth muscle cells has not been confirmed.

The identity and amount of protein synthesis are critical parameters for determining the ribosomal functional state [22]. As a vascular proliferative disease, neointimal hyperplasia is accompanied by substantial nascent protein synthesis. Puromycin (puro) is an aminonucleoside antibiotic, which covalently bonds to the C terminus of the nascent polypeptide chain, causing the premature termination of nascent polypeptide chains that block the protein synthesis [23]. Detecting nascent polypeptide-puro conjugates with anti-puro antibodies can effectively detect the nascent proteins.

The present study explores the correlation between BOP1 and neointimal formation. We first detected the mRNA and protein expression levels of BOP1 in the neointimal or normal tissue of human coronary specimens. Then, we used the mouse model of carotid artery ligation and rat model of carotid artery balloon injury that stimulated the pathological changes after PCI to investigate whether BOP1 was elevated in neointimal hyperplasia and its effects on proliferation of VSMCs. Next, we studied the cellular mechanisms by evaluating the impacts of BOP1 knockdown on proliferation, migration, cell cycle, and apoptosis of human vascular smooth muscle cells (HVSMCs). Finally, we further explored the intrinsic molecular mechanisms that mediated the effects of BOP1 knockdown.

## 2. Materials and Methods

### 2.1. Human Tissue Samples

Coronary artery samples were collected from patients who were diagnosed with CAD and received a heart transplant operation in Renmin Hospital of Wuhan University. Normal coronary artery specimens were collected from patients without CAD who served as donors of heart transplant surgery in Renmin Hospital of Wuhan University. The present study was approved by the Ethics Committees of Renmin Hospital of Wuhan University of China, and all procedures involved in the human samples strictly abided by the Declaration of Helsinki. An informed written consent was obtained from all subjects. The clinical characters of the patients enrolled in this study are summarized in Supplementary Table 1. The coronary arteries of humans were deprived from the heart after the heart transplant operation. The coronary artery was irrigated for several times with precooled saline and divided into two parts. One part was collected into a cryopreservation tube and stored in liquid nitrogen, while the other part was fixed with 4% paraformaldehyde (PFA; Servicebio, #G1001-500ML, China) and embedded in paraffin for the next analysis.

### 2.2. Animals

The p53 knockout mice (B6.129S2-Trp53tm1Tyj/JNju) were purchased from Jackson Laboratory (USA) and were crossed to obtain the homozygous, heterozygote, and wild type. The wild-type littermates served as the control group. The primers used for genotype identification of mice were synthesized by Sangon Biotech (Shanghai, China), and sequences are as follows: wild-type p53 gene Forward: 5′-ACAGCGTGGTGGTACCTTAT-3′, Reverse: 5′-TATACTCAGAGCCGGCCT-3′ and mutant p53 gene Forward: 5′-CTATCAGGACATAGCGTTGG-3′, Reverse: 5′-TATACTCAGAGCCGGCCT-3′. Merely male 12-week old mice were used for the present research. All procedures performed on the animals were supported by the Institutional Animal Care and Use Committee of Wuhan University. The experiments were designed in accordance with the Guide for the Care and Use of Laboratory Animals. All animal experiments were carried out in the Cardiovascular Surgery Laboratory, Renmin Hospital of Wuhan University.

The mouse model of carotid artery ligation and rat model of carotid artery balloon injury were established according to standard instructions. Briefly, after pentobarbital sodium anesthesia, hair removal and neck midline incision were performed, and the left carotid artery and its branches were carefully separated. For the rat balloon injury model, a 1.25 F balloon (balloon diameter 1.25 mm, balloon length 1 mm) with a guide wire (Medtronic, # SPL12515X, USA) was inserted into the common carotid artery from the external carotid incision. Then, the catheter was inflated to expand the carotid artery. After the balloon was withdrawn, the external carotid artery was ligated at the proximal end of the incision near the bifurcation, and the blood flow was restored. For the mouse artery ligation model, a 5-0 silk suture ligation of the distal end of the left common carotid artery near the bifurcation was used to induce the vascular remodeling.

For testing the mechanism of BOP1 in the neointimal formation, the lentivirus of LV-BOP1 and LV-SCR (scrambled sequences) was loaded in F-127 (Sigma, #P2443-250G, USA) gel supplemented with 0.25% trypsin (Servicebio, #G5030-5G, China) and applied around the left carotid artery after ligation. The carotid arteries were harvested at 7, 14, and 28 days after ligation. The animal was anesthetized, and the heart was perfused with physiological saline before harvesting the carotid artery. Then, the mouse carotid arteries were fixed with 4% PFA solution and embedded in paraffin for sectioning. Serial cross-sections (5 *μ*m) were cut for further analysis.

### 2.3. Cell Culture and Transfection

The human vascular smooth muscle cell line (HVSMC) (ATCC® PCS-100-012™) was purchased from the China Centre for Type Culture Collection. The HVSMCs were maintained at 37°C in a humidified atmosphere that contained 5% CO_2_ and cultured with high-glucose DMEM (Genom, #GNM12800, China) that contained 10% fetal bovine serum (FBS) (Gibco, #10100, USA) and 1% penicillin and streptomycin solution (Servicebio, #G4003-100ML, China). Lentivirus loading BOP1 RNAi sequence (LV-BOP1; # PIEL248033814) including RNAi-1 (60973-1): TAGCAAGCTGGTGTGGTTT, RNAi-2 (60974-1): CCACAAGATGCACGTACCT, and RNAi-3 (60975-1): TGGAGTGGTACGATGACTT and related scrambled (LV-SCR; # LVCON077): TTCTCCGAACGTGTCACGT were constructed by GeneChem Group (Shanghai, China). HVSMCs in logarithmic growth were transfected with LV-BOP1 or LV-SCR at multiplicity of infection (MOI) = 40 for 12 h. The cells were further cultured in medium with 10% FBS for 3 d. For RPL11 knockdown, HVSMCs were plated the day before siRNA transfection. A nontargeting siRNA sequence (NC siRNA) (RiboBio, # S1012, China) or human RPL11-specific siRNA (RPL11 siRNA) (genOFFTM st-h-RPL11-003; sequence: ACCCAAGCATTGGTATCTA; RiboBio, # T1022, China), purchased from RiboBio (Guangzhou, China), were added at 50 nM by using riboFECTTM CP Transfection Kit (RiboBio, # C10511-05, China) according to the manufacturer's instructions. The media was changed to fresh media 24 h after transfection. Cells were incubated with the corresponding drugs for additional detection.

### 2.4. Quantitative Real-Time PCR

According to the general instructions, the total RNA were extracted by TRIzol reagent (Takara, #9108, Japan), and the purity and concentration of RNA were determined using NanoDrop (Thermo Fisher, USA). Then, the reverse transcription kit (Yeasen, #11119ES60, China) was used to reverse transcribe the total RNA into cDNA. Real-time PCR was performed using the qPCR SYBR Green Master Mix (Yeasen, #11119ES60, China) in the Bio-Rad PCR system (Bio-Rad, USA). The primers were synthesized by Sangon Biotech (Shanghai, China), and sequences are as follows: BOP1 Forward: 5′-GCCACAAGATGCACGTACCT-3′, Reverse: 5′-TTCCTGGATGAAGCGTCCGTA-3′ (Accession: NM_015201.5); Ki-67 Forward: 5′-ACGCCTGGTTACTATCAAAAGG-3′, Reverse: 5′-CAGACCCATTTACTTGTGTTGGA-3′ (Accession: NM_ 001145966.2); and *β*-actin Forward: 5′-CATGTACGTTGCTATCCAGGC-3′, Reverse: 5′-CTCCTTAATGTCACGCACGAT-3′ (Accession: NM_001101.5). The absence of nonspecific results by blasting the primers was verified. The expression of BOP1 and Ki-67 was normalized to the level of *β*-actin in the same cDNA. The 2^-*∆∆*Ct^ method was used to compare the mRNA levels.

### 2.5. Western Blot

Coronary artery tissues or cells were washed with cold PBS. Then, the total protein was harvested using RIPA buffer (Servicebio, #G2002-100ML, China) containing the 1% protease inhibitor cocktail (1 : 50, Servicebio, #G2006-250UL, China) and phenylmethylsulfonyl fluoride (PMSF, 1 : 100; Servicebio, #G2008-1ML, China) and centrifuged at 12,000 rpm for 15 minutes at 4°C. The supernatant was carefully sucked out and ultrasonication was performed; the protein concentration of the lysate was detected using the BCA Protein Quantitation Kit (Beyotime Bio, #P0012, China). Then, 120 *μ*g total protein of the human samples or 50 *μ*g total protein of cells were loaded into the 8%-12% SDS-PAGE gel for western blot assay, based on standard procedures. To inhibit protein synthesis, 50 *μ*g/ml cycloheximide (CHX; Beyotime Bio, # SC0353-5 mg, China) was treated for 0 min, 30 min, 60 min, and 90 min before testing. The nascent proteins were detected using the anti-puromycin antibody (1 : 1,000, Merck Millipore, #MABE343-AF488, Germany), and the gels were stained with Coomassie blue (Servicebio, #GM1002, China).

### 2.6. EVG and Immunofluorescence Staining

In order to measure the area of neointima in the mouse carotid artery after ligation injury and human coronary artery, all samples were collected and fixed with 4% PFA at room temperature for 24 hours, embedded in paraffin, and sectioned. The sections were stained with an elastic van Gieson (EVG) stain (Servicebio, #XH184602, China) using previously published protocols [24, 25]. All images were observed under an optical microscope (BX6, Olympus, Japan). The average of 3 equidistant segments were taken from each section, using six EVG sections from each group. The intima area was calculated as the internal elastic lamina area minus the luminal area, and the medial area was calculated as the external elastic lamina area minus the internal elastic lamina area. Intima/media ratio was then calculated.

The immunofluorescence double staining detected the colocalization of BOP1 and Ki-67 in neointima, in order to assess the connection between BOP1 and VSMCs proliferation. The antigen of the slices were permeabilized with 0.2% Triton X-100 (Servicebio, #WGT8200, China), restored by microwave heating, and blocked with 5% goat serum (Beyotime Bio, #C0265, China). Then, these were incubated overnight with anti-BOP1 (1 : 200, Bioss, #bs-12877R, China) and Ki-67 (1 : 100, Bioss, #bsm-33070 M, China), or for other purposes with anti-PCNA (1 : 100, Servicebio, #GB14139, China), anti-MDM2 (1 : 50, Santa Cruz Biotechnology, #sc-965,USA), and anti-RPL11 (1 : 100, Proteintech, #16277-1-AP, China). Afterwards, these were incubated with FITC-labeled goat anti-mouse (1 : 50, Servicebio, #GB22301, China) and Cy3-labeled goat anti-rabbit IgG (1 : 100, Servicebio, #GB21303, China), and subsequently counterstained with 4′,6-diamidino-2-phenylindole (DAPI, 2 *μ*g/ml; Servicebio, #G1012-10ML, China) for five minutes. Fluorescence microscopy (Olympus BX6 with DP72 Camera, Japan) was employed to capture the image. All images were quantified using the ImageJ software. Five visual fields were randomly selected of each sample, and the fluorescence intensity or average number of positive cells were calculated.

### 2.7. Cell Viability Assay

HVSMCs cultured for 3 days after conducted relevant genetic manipulation and pretreated with 10 *μ*M of the specific p53 inhibitor PFT-*α* (Selleck, # S2929, USA) or equal dimethyl sulfoxide (DMSO; Servicebio, # WGD2650, China) for 12 h were seeded in 96-well plates at the density of 3 × 10^3^/190 *μ*l/well; each group had three repetitions. After continuing to treat with the same concentration of PFT-*α* or DMSO for the corresponding time, 10 *μ*l of Cell Counting Kit-8 (CCK8) detection reagent (Beyotime Bio, #C0039, China) was added to each well and the optical density at 450 nm of each well was measured by microplate reader (Perkin Elmer, USA). The cell viability was determined every 12 hours for the next 3 days as described.

### 2.8. Detection of Cell Migration by the Wound Healing Assays

To study the role of p53 on migration capacity change, HVSMCs were pretreated with 10 *μ*M PFT-*α* or DMSO followed by resuspended and cotreated with 20 ng/ml platelet-derived growth factor-BB (PDGF-BB; MedChemExpress, # HY-P7055, China). The ability of cell migration was evaluated via wound healing assay as our previous described [13].

### 2.9. Detection of Cell Migration by the Transwell Assay

HVSMCs pretreated with 10 *μ*M PFT-*α* or DMSO at a density of 2 × 10^4^ cells in 180 *μ*l serum-free medium were planted in the upper chamber of a Transwell apparatus (Corning, #3374, China), and 800 *μ*l complete medium was placed into the lower chamber. After incubation for 12 hours, the upper chamber was removed and cells that had not migrated were gently wiped off with a swab. After washing and fixing with 4% PFA, the Transwell membranes were stained with 0.1% crystal violet (Servicebio, #G1014-50ML, China) for 15 min. Images were captured using a light microscope (BX51, Olympus, Japan). Cells were counted at a magnification of ×400 in 5 randomly selected fields from each chamber.

### 2.10. Quantification of Protein Synthesis

The rate of protein synthesis was measured, as previously described [13].

### 2.11. Flow Cytometric Analysis

For the analysis of cell cycle progression, HVSMCs were centrifuged at 800 rpm for 5 min and the supernatant was discarded. The cells were resuspended in 250 *μ*l of PBS and 750 *μ*l of anhydrous ethanol and fixed at -20°C for 24 h. The fixed samples were centrifuged at 1500 rpm for 5 min, and the supernatant was removed. The cells were washed twice with 1 ml of precooled PBS and labeled with 500 *μ*l PI/RNase Staining Buffer (Biosciences, #550825, USA) in the dark at room temperature for 15 min. Cell doublets were excluded by gating on the fluorescence width versus area plot, and the proportion of cells in each phase of the cell cycle was determined by flow cytometry. The cytometric analysis of cell apoptosis and detection of reactive oxygen species (ROS) by flow cytometry were conducted, as previously described [13]. Data acquisition was executed using CytoFLEX (BC43326, Beckman, USA) and further analyzed with FlowJo software.

### 2.12. Statistical Analysis

All quantitative data in the present study were presented as mean ± standard deviation (SD), and the statistical analysis was performed using the GraphPad Prism 7.0 software. All data were tested for normality and equal variance. If the data passed these tests, the two-group comparison was evaluated by Student's *t*-test and one-way ANOVA, followed by Tukey's post hoc test for multiple comparisons. If the data did not pass these tests, Mann–Whitney test was performed to compare the two groups. *P* < 0.05 was considered statistically significant. Each experiment was repeated for at least three times.

## 3. Results

### 3.1. BOP1 Significantly Upregulated in VSMCs during Neointimal Hyperplasia

**Figure 1:**
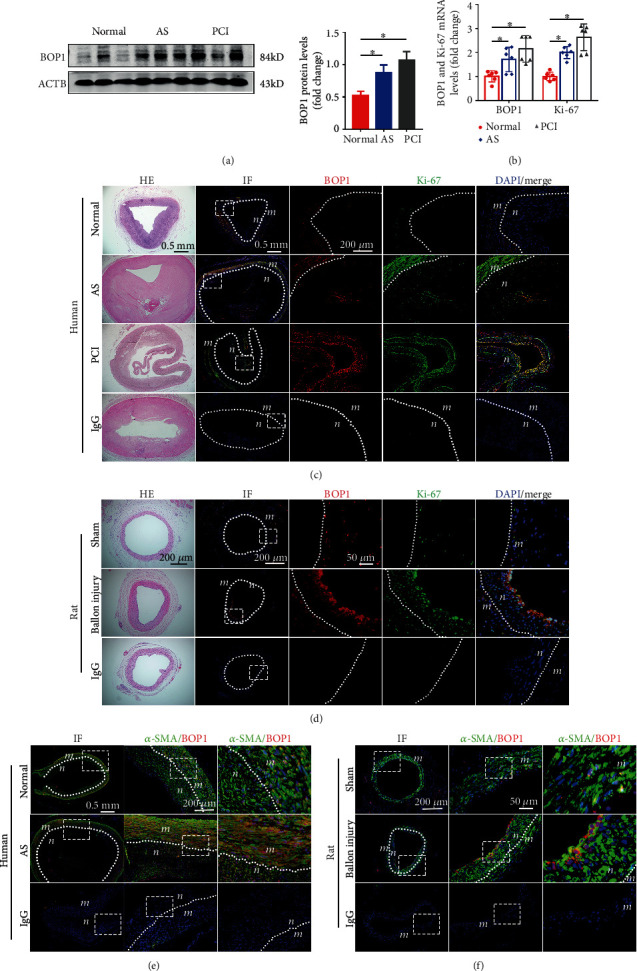
BOP1 significantly upregulated in VSMCs during neointimal hyperplasia in human coronary arteries. (a, b) Western blot and qRT-PCR were used to detect the expression of BOP1 in normal (normal), atherosclerosis (AS), and restenosis after the percutaneous coronary intervention (PCI) in human coronary samples (*n* = 6). (c) Double immunofluorescence (IF) staining was performed to detect the expression of BOP1 (red) and Ki-67 (green) in normal (Normal), atherosclerosis (AS), and restenosis after the percutaneous coronary intervention (PCI) in human coronary samples. The isotype-specific IgG was used as the negative control group. The nuclei were stained with DAPI (blue) (*n* = 3). The white dotted lines indicated the boundary between the neointima (n) and media (m). (d) BOP1 (red) and Ki-67 (green) expressions were detected in the carotid artery of Sprague-Dawley rats that underwent balloon injury (balloon injury) or the sham group (sham) via double immunofluorescence. The cell nuclei were stained with DAPI (blue) (*n* = 3). (e, f) The immunofluorescence staining of *α*-SMA (green) and BOP1 (red) in normal/AS human coronary samples or balloon injury/sham rats carotid arteries. The nuclei were stained with DAPI (blue) (*n* = 3). The representative images are shown. The data were described as mean ± standard deviation (SD). ^∗^*P* < 0.05 was determined by one-way ANOVA, followed by Tukey's post hoc test.

In order to test the present hypothesis, the investigators initially examined the expression of BOP1 in the coronary artery samples. The western blot and qRT-PCR revealed that BOP1 was highly expressed in the coronary artery with atherosclerosis (AS), or the coronary artery obtain from patients who received percutaneous coronary intervention (PCI) therapy, at the protein and mRNA level (Figures 1(a) and 1(b)).

The immunofluorescence staining revealed that BOP1 and Ki-67 were significantly upregulated, and the positive rate of colocation of BOP1 and Ki-67 was significantly elevated in the coronary artery harvested from patients with severe coronary obstructions or restenosis after PCI, compared with normal coronary arteries (Figure 1(c)). This means that BOP1 may be closely correlated with VSMC proliferation in the neointimal formation. In order to further confirm these findings, rat carotid artery balloon injury models were constructed. The immunofluorescence double staining revealed that BOP1 and Ki-67 were also markedly upregulated and colocalized at the neointima in rat carotid arteries after injury (Figure 1(d)). In order to further determine whether BOP1 was mainly expressed in VMSCs, double immunofluorescence stain was performed to detect the expression of alpha-smooth muscle actin (*α*-SMA) and BOP1. As expected, BOP1 was mainly expressed in VSMCs in both human coronary samples and rat carotid arteries (Figures 1(e) and 1(f)).

### 3.2. BOP1 Knockdown Suppresses Neointimal Hyperplasia *In Vivo*

**Figure 2:**
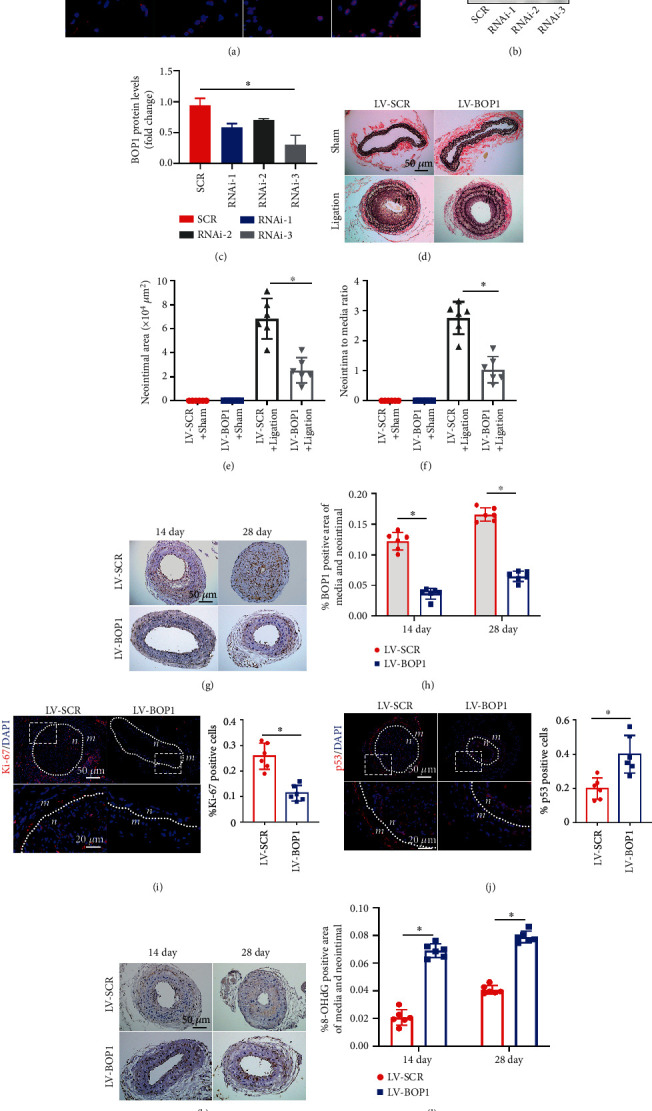
BOP1 knockdown suppresses neointimal hyperplasia *in vivo.* (a) The immunofluorescence staining shows the BOP1 (red) location and expression in the carotid artery at different time points after ligation. The views in the white dotted box in the upper panels were magnified and presented in the lower panels. The nuclei staining with DAPI (blue) (*n* = 5). (b, c) The representative protein western blot bands of VSMCs transfected with the lentivirus loaded with the BOP1 RNAi sequence or scrambled sequences (*n* = 3). (d) The EVG staining sections exhibit the carotid artery structures from the carotid artery ligation model (ligation) or sham group (sham) treated with LV-BOP1 or LV-SCR at 28 days after ligation (*n* = 6). (e, f) The neointimal area and neointima-to-media ratio were measured at six cross-sections, and the means were calculated. (g, h) The immunohistochemical staining and percentage of the area positive for BOP1 in the media and neointimal after carotid artery transfection with LV-BOP1 or LV-SCR for 14 or 28 days. (i) Representative immunofluorescence stain images and the rate of Ki-67-positive (red) cells in the media and neointimal after transfection with the LV-BOP1 or LV-SCR (*n* = 6). (j) p53-positive (red) cells in the media and neointimal after transfection with LV-BOP1 or LV-SCR. (k, l) Immunohistochemical staining and the percentage of the area positive for 8-OHdG in the media and neointimal after carotid artery transfection with LV-BOP1 or LV-SCR for 14 or 28 days (*n* = 6). The representative images are shown. The data were presented as the mean ± standard deviation (SD); ns means that there is no statistical significance, ^∗^*P* < 0.05; one-way ANOVA followed by Tukey's post hoc test for (c) and Student's *t*-test for (e, f, h–j, l).

At 0, 14, 21 and 28 days after ligation, it was observed that BOP1 was mainly expressed on the inside edge of the neointima of the mouse carotid artery (Figure 2(a)). In order to compare the efficiency of these three types of RNAi, the lentivirus loaded with the BOP1 RNAi sequence and scrambled sequences were transfected in HVSMCs. The protein expression levels of BOP1 significantly decreased in the RNAi group, compared with the scrambled sequences group (LV-SCR). The lentivirus loaded with RNAi-3 (LV-BOP1) had the highest knockdown efficiency and was used in the subsequent experiments (Figures 2(b) and 2(c)). The LV-BOP1 was loaded in F-127 with 0.25% trypsin and applied around the mouse carotid artery. At 28 days after ligation, the EVG stain revealed that the LV-BOP1 potently restrained the neointimal formation, when compared with the LV-SCR control group (Figure 2(d)). Furthermore, the neointimal area and neointima-to-media ratio in the LV-BOP1 group also significantly decreased, compared with the LV-SCR group (Figures 2(e) and 2(f)). The immunohistochemical staining confirmed that compared with the LV-SCR group, the percentage of the area positive for BOP1 in the media and neointimal of the carotid artery in the LV-BOP1 group significantly decreased at 14 and 28 days after ligation, respectively (Figures 2(g) and 2(h)). The carotid artery immunofluorescence staining of Ki-67 revealed that the rate of Ki-67-positive cells in the media and neointimal significantly decreased after transfected with the LV-BOP1, when compared with the LV-SCR-transfected counterparts (Figure 2(i)). p53 is an important proliferation-related regulatory molecule and also a vital protein of nucleolar stress signaling pathway. The immunofluorescence revealed that p53-positive cells in the media and neointimal significantly increased after BOP1 knockdown in the ligated mouse carotid artery, compared with the LV-SCR group (Figure 2(j)). Our previous study depicted that overexpression of BOP1 attenuated ROS production and cell apoptosis under serum-free and hypoxic conditions of HVSMCs in a p53-dependent way [13]. We therefore detected 8-hydroxy-2-deoxyguanosine (8-OHdG), a biomarker indicated the DNA damage induced by ROS, in the ligated carotid artery. Compared with the LV-SCR group, LV-BOP1 raised 8-OHdG which may damage HVSMCs and contribute to mitigating neointimal hyperplasia (Figures 2(k) and 2(l)). All these above suggest that BOP1 may have accelerated the neointimal formation, and p53 may have played a vital role in this process.

### 3.3. BOP1 Deficiency Restrains Neointimal Hyperplasia by Upregulating the Expression of p53

**Figure 3:**
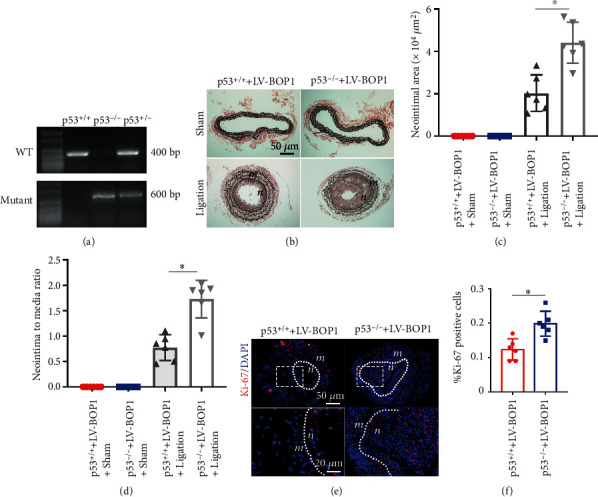
BOP1 deficiency restrains neointimal hyperplasia by upregulating the expression of p53. (a) Total DNA extracted from mouse tails were collected and subsequently performed with agarose gel electrophoresis for determining genotype of p53 knockout mice (p53^−/−^), p53 partially knockout mice (p53^-/+^), and wildtype littermates(p53^+/+^). WT: wild-type p53 gene, mutant: p53 knockout gene. (b) The EVG staining sections exhibited the carotid artery structures from p53^−/−^ or p53^+/+^ mice treated with LV-BOP1 at 28 days after carotid artery ligation (ligation) or the sham group (sham) (*n* = 6). (c, d) The neointimal area and neointima-to-media ratio were measured at six cross-sections, and the means were calculated. (e, f) The immunofluorescence staining of Ki-67 (red) and the positive cell rate of the neointima after transfection with LV-BOP1 in p53^−/−^ mice or p53^+/+^ group (*n* = 6). The representative images are shown. The data were represented as the mean ± standard deviation (SD); ^∗^*P* < 0.05 determined by Student's *t*-test.

In order to investigate the role of p53 in BOP1 suppression on neointimal formation, we constructed the p53 knockout mice. Agarose gel electrophoresis was used to identify the homozygous p53 deletion mice. The band of p53^**-/-**^ was located at 600 bp but was absent at 400 bp (Figure 3(a)). The inhibitory effect of LV-BOP1 on the neointimal hyperplasia was partly reserved in p53^**-/-**^ mice (Figure 3(b)). The statistical results revealed that the area of the neointima (Figure 3(c)) and the ratio of the intima to the media (Figure 3(d)) in the p53^−/−^+LV-BOP1 group increased, when compared with p53^+/+^+LV-BOP1. In addition, the Ki-67-positive cell rate increased in the p53^−/−^+LV-BOP1 group, compared with p53^+/+^+LV-BOP1 (Figures 3(e) and 3(f)). Hence, the p53 knockout *in vivo* partially reversed antiproliferation of LV-BOP1 in the ligation-induced neointima hyperplasia. These above results indicate that BOP1 deficiency restrains the neointimal hyperplasia mainly by upregulating the expression of p53.

### 3.4. p53 Activation Mediated the Biological Effects of LV-BOP1 on HVSMCs

**Figure 4:**
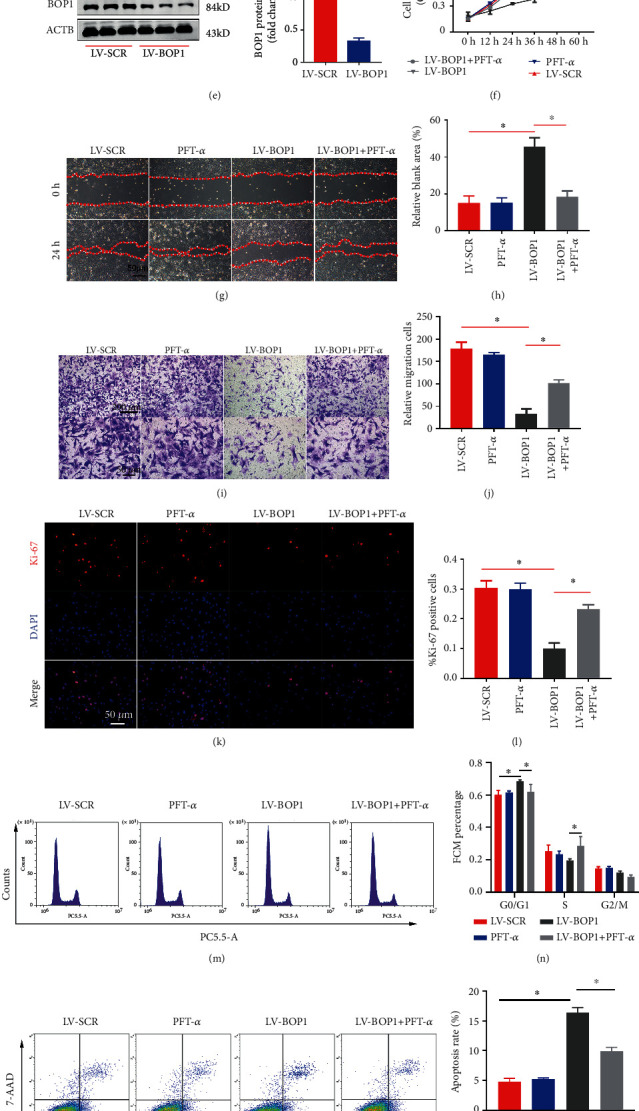
p53 activation mediated the biological effects of LV-BOP1 on HVSMCs. (a, b) mRNA and protein expressions of BOP1 after stimulation with different doses of PDGF-BB (*n* = 3). (c, d) mRNA and protein expressions of BOP1 after stimulation of 20 ng/ml PDGF-BB at different times (*n* = 3). (e) BOP1 expression in HVSMCs after transfection with LV-BOP1 or LV-SCR detected by western blot (*n* = 3). (f) CCK-8 assay detecting the HVSMC growth rates of BOP1 knockdown and cotreated with PFT-*α* (10 *μ*M) or not (*n* = 3, ^∗^*P* < 0.05, the LV-BOP1 group compared with the LV-SCR group) (*n* = 3, ^&^*P* < 0.05, the LV-BOP1 group compared with the LV-BOP1+PFT-*α* group). (g–j) HVSMCs transfected with LV-SCR or LV-BOP1 followed by pretreatment with 10 *μ*M PFT-*α* or equal DMSO for 12 h. HVSMCs were resuspended and cotreated with 20 ng/ml PDGF-BB and 10 *μ*M PFT-*α* or equal DMSO for the scratch test. Phase-contrast microscopy images showed the migratory capacity of VSMCs under different treatment. Transwell assay was performed to assess HVSMC migration. Diagram showed quantification of cell migration rate over time. (*n* = 3),^∗^*P* < 0.05. (k, l) The Ki-67 (red) immunofluorescence staining revealed that the BOP1 knockdown markedly restrained the HVSMC proliferation, while PFT-*α* (10 *μ*M) partly reversed the inhibit effect induced by the BOP1 knockdown, (*n* = 3). (m, n) HVSMCs transfected with LV-BOP1 or LV-SCR, then pretreated with 10 *μ*M PFT-*α* or equal DMSO for 12 h. The proportion of cells in each phase of the cell cycle was determined by flow cytometry (n = 3). (o, p) Apoptosis was assessed by Annexin V-PE/7-AAD staining and flow cytometry. (q, r) ROS were detected by 2′,7′-dichlorodihydrofluorescein diacetate (DCFH-DA) and flow cytometry (*n* = 3). Data are represented by mean ± SD. ^∗^*P* < 0.05; one-way ANOVA followed by Tukey's post hoc test for (a–f, h, j, l, n, p, r) and Student's *t*-test for (e).

Due to the efficiency of VSMC mitogen, PDGF-BB have been widely used in VSMC study, which strongly facilitates VSMC proliferation and migration [26, 27]. In order to explore the underlying connections between BOP1 and cell proliferation and migration, we analyzed the expression of BOP1 at the mRNA and protein levels by qPCR and western blot, after stimulating HVSMCs with PDGF-BB. Intriguingly, BOP1 significantly increased in a dose- and time-dependent manner in HVSMCs after PDGF-BB administration (Figures 4(a)–4(d)). Therefore, BOP1 may be closely correlated to the proliferation of VSMCs *in vitro*.

Next, we constructed BOP1 knockdown HVSMC lines, and the knockdown efficiency of LV-BOP1 was approximately 70% (Figure 4(e)). The CCK-8 assay and Ki-67 immunofluorescence staining were used to evaluate the proliferation ability. LV-BOP1 markedly decreased the proliferation ability of HVSMCs, compared with the LV-SCR group, and was partly restored by pretreatment with PFT-*α* (Figures 4(f), 4(k), and 4(l)). In addition, the number of cell migration significantly decreased after BOP1 knockdown in HVSMCs, compared with the LV-SCR group, and was also partly reversed by PFT-*α* (Figures 4(g)–4(j)), as determined by the wound healing and Transwell assay. Flow cytometry confirmed that BOP1 knockdown blocked HVSMC cell cycle in the G0/G1 phase, and PFT-*α* partially reversed this effect (Figures 4(m) and 4(n)). What is more, BOP1 knockdown influenced the redox balance, elevated HVSMC ROS production and apoptosis, and could be partly reversed by PFT-*α*, as detected by flow cytometry (Figures 4(o)–4(r)). The present data suggest that BOP1 knockdown could significantly affect the biological function of HVSMCs, through a way of p53 expression regulation.

### 3.5. RPL11 Knockdown Partly Reserved the Biological Effects of LV-BOP1 on HVSMCs

As has been extensively demonstrated in other cell lines, RPL11 is of great importance in the p53-dependent nucleolar stress response pathway [28, 29]. We therefore explored whether RPL11 is a downstream target of BOP1 knockdown.

**Figure 5:**
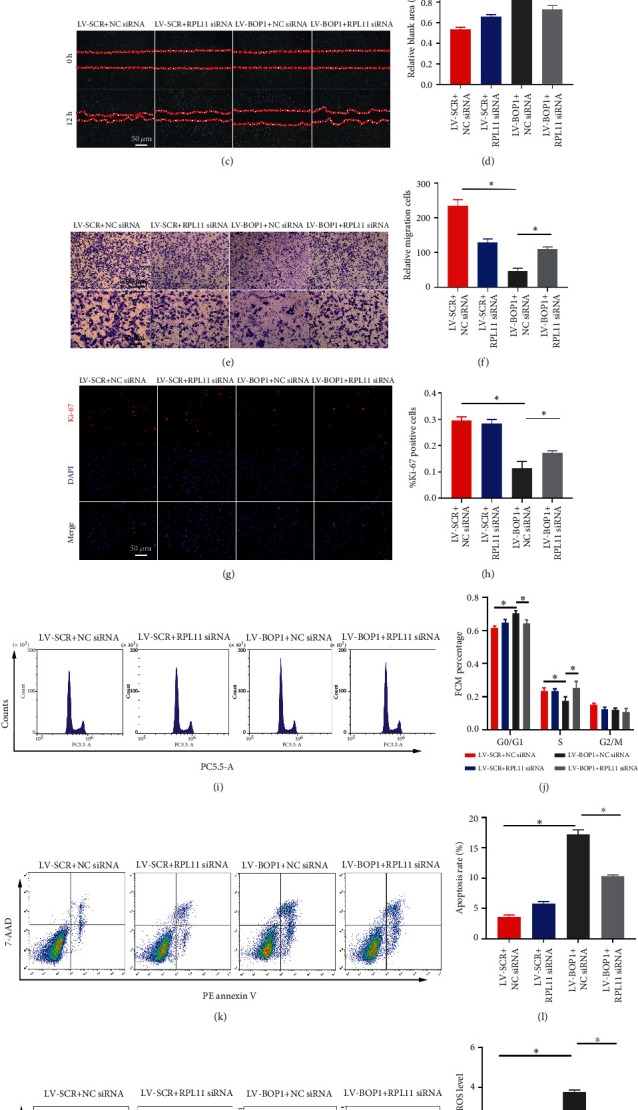
RPL11 knockdown partly reserved the biological effects of LV-BOP1 on HVSMCs. (a, b) The representative western blot bands and relative expression levels of RPL11 in HVSMCs after transfection with NC siRNA or RPL11 siRNA (*n* = 3). (c–f) HVSMCs transfected with LV-SCR or LV-BOP1 followed by 50 nM NC siRNA or RPL11 siRNA pretreatment for 24 h. HVSMCs were resuspended and cotreated with 20 ng/ml PDGF-BB for the scratch test. Images were captured by phase-contrast microscopy. Diagram showed quantification of cell migration rate over time. Transwell assay was performed to assess HVSMC migration. The quantification of migrated cells through Transwell chamber was presented. (*n* = 3), ^∗^*P* < 0.05. (g, h) The representative Ki-67 (red) immunofluorescence staining images (*n* = 3) and the positive cell rate of HVSMCs transfected with LV-SCR or LV-BOP1 followed by 50 nM NC siRNA or RPL11 siRNA were presented. (*n* = 3), ^∗^*P* < 0.05. (i, j) HVSMCs transfected with LV-BOP1 or LV-SCR were treated with 50 nM NC siRNA or RPL11 siRNA. The proportion of cells in each phase of the cell cycle was determined by flow cytometry (*n* = 3). (k, l) Apoptosis was assessed by Annexin V-APC/7-AAD staining and flow cytometry (*n* = 3). (m, n) ROS were detected by DCFH-DA and flow cytometry (*n* = 3). Data are represented by mean ± SD. ^∗^*P* < 0.05; one-way ANOVA followed by Tukey's post hoc test for (d, f, h, j, l, n) and Student's *t*-test for (b).

The knockdown efficiency of RPL11 siRNA-3 validated by western blot in HVSMCs was approximately 50% and was used in subsequent studies (Figures 5(a) and 5(b)). The constructed cell lines transfected with LV-SCR or LV-BOP1 of HVSMCs were subsequently treated with NC siRNA or RPL11 siRNA. As detected by Ki-67 immunofluorescent staining and the wound healing and Transwell assay, both the decreased proliferation and migration ability of LV-BOP1-transfected HVSMCs were partly reserved by RPL11 siRNA (Figures 5(g) and 5(h) and 5(c)–5(f)). Flow cytometry also confirmed that the G0/G1 cell cycle arrest, ROS production, and cell apoptosis were partly reserved by RPL11 siRNA (Figures 5(i)–5(n)). These results together suggested that the biological effects of LV-BOP1 in HVSMCs were at least partly mediated by RPL11.

### 3.6. Knockdown BOP1 Suppresses HVSMC Migration and Proliferation through RPL11/MDM2/p53 Nucleolar Stress Response Pathway and Protein Synthesis Inhibition

BOP1 is mainly involved in the maturation of the pre-rRNA and protein synthesis rate [19, 20]. Restraining the physiological function of BOP1 resulted in the accumulation of free ribosomal protein L5, L11, and L23, which in turn negatively regulated the MDM2 activity and downregulated the rate of protein synthesis [30, 31]. Nevertheless, ribosomal biogenesis and proteinic synthesis were necessary for the cell proliferation and migration [32, 33]. Overall, it was speculated that suppressing BOP1 decreases the migration and proliferation of HVSMCs via the activation of the RPL11/MDM2/p53 nucleolar stress signal pathway, thereby slowing down the rate of nascent protein synthesis.

As expected, BOP1 knockdown decreased the rate of nascent protein synthesis *in vitro* and could not be restored by PFT-*α* (Supplementary Figure S1A and S1B). Therefore, the downregulation of the nascent protein synthesis rate may be due to the individual element of BOP1 deletion of the HVSMC biological function.

**Figure 6:**
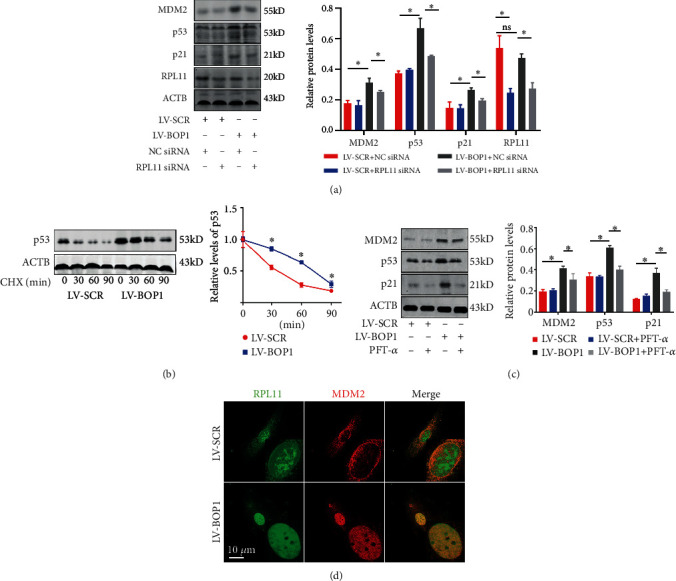
BOP1 knockdown activated RPL11/MDM2/p53 nucleolar stress response pathway. (a) The representative western blot bands and relative expression levels of MDM2, p53, p21, and RPL11 in LV-BOP1 or LV-SCR transfected HVSMCs followed by 50 nM NC siRNA or RPL11 siRNA treatment (*n* = 3). (b) HVSMCs were transfected with LV-BOP1 or LV-SCR and administrated with CHX at different time points for detecting the p53 degeneration rate by western blot (*n* = 3). (c) Western blot analyzed the expression of MDM2, p53, and p21 in LV-BOP1 or LV-SCR transfected HVSMCs, with or without PFT-*α* treatment (*n* = 3). (d). Confocal microscopy was used to detect the expression and location of RPL11 (green) and MDM2 (red) after the HVSMC transfection with LV-BOP1 or LV-SCR (*n* = 6). The data are presented as the mean ± standard deviation (SD). ns means that there is no statistical significance, ^∗^*P* < 0.05, determined by one-way ANOVA, followed by Tukey's post hoc test.

The ubiquitination and degeneration of p53 were mainly induced by MDM2 [33, 34]. RPL11 plays an important role in inhibiting the MDM2 function through the direct connection with the zinc finger of MDM2 [35, 36]. Therefore, RPL11 may inhibit MDM2-mediated p53 ubiquitination by binding to MDM2. After BOP1 deletion, MDM2, p53, and p21 were markedly upregulated in HVSMCs, while knockdown RPL11 by siRNA or inhibit p53 by PFT-*α* reserved the upregulation to varying degrees (Figures 6(a) and 6(c)). In order to determine the change in p53 ubiquitination and degeneration after BOP1 knockdown, HVSMCs were treated with 50 *μ*g/ml of CHX for 0, 30, 60, and 90 minutes before testing. The degeneration rate of p53 significantly decreased after the BOP1 knockdown (Figure 6(b)). As the autoregulatory feedback loop is probably needed to maintain a critical MDM2/p53 ratio within a cell and MDM2 is known to self-ubiquitinate and may lead to degradation [29, 37], we speculate that the upregulation of MDM2 might have been induced by the decreased MDM2 self-ubiquitination. The immunofluorescence staining confirmed that MDM2 was mainly located at the nucleus, and RPL11 was mainly positioned at the nucleoli in HVSMCs. After BOP1 knockdown, the binding of endogenous RPL11 to MDM2 was much greater than that in the control group (Figure 6(d)). These results indicate that RPL11 translocated from the nucleoli to the nucleoplasm and combined with MDM2, which might be the possible mechanism for both p53 activation and MDM2 inactivation.

**Figure 7:**
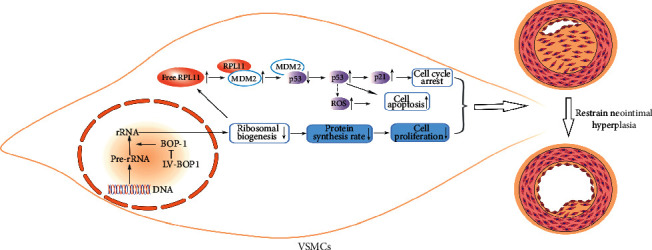
Graphics summarizes the roles of BOP1 knockdown in regulation of neointimal hyperplasia. Downregulating BOP1 by lentivirus, on the one hand, leads to the accumulation of free RPL11 in cytoplasm, and then RPL11 combines with MDM2, which restrains MDM2-mediated degeneration of p53, subsequently inducing p53-mediated cell cycle arrest and apoptosis; on the other hand, BOP1 knockdown impairs rRNA maturation and ribosome biogenesis and deregulates the rate of nascent proteins synthesis, thereby inhibiting neointimal hyperplasia. BOP1: blocking of proliferation 1; RPL11: ribosomal protein L11; MDM2: murine double minute 2; VSMCs: vascular smooth muscle cells.

In general, activation of the RPL11/MDM2/p53 nucleolar stress response pathway and the decrease of nascent protein synthesis rate might be the independent regulator for the suppression of VSMC proliferation and migration abilities after the BOP1 knockdown (Figure 7).

## 4. Discussion

In the present study, the following were revealed: (i) BOP1 is highly expressed on the inside edge of the neointima in human coronary arteries with atherosclerosis and restenosis, as well as in arteries of animal neointimal hyperplasia models. (ii) BOP1 knockdown subsequently inspired the RPL11/MDM2/p53 nucleolar stress response pathway and inhibited the nascent protein synthesis, which in turn inhibited the proliferation and migration of HVSMCs *in vitro* and neointimal hyperplasia *in vivo*. As far as it is known, this is the first time that BOP1 has been found closely related to neointimal hyperplasia and knockdown BOP1 may have profound implications for the treatment of neointimal hyperplasia.

The tight connection between BOP1 and cell proliferation has been demonstrated in several researches [35, 36]. In addition, BOP1 was also found to affect the cell migration in hepatoma [12]. VSMC migration and proliferation are the pivotal events in neointimal hyperplasia during artery restenosis and atherosclerosis [38]. In the present study, it was found that BOP1 is highly expressed in VSMCs during neointimal hyperplasia *in vivo* and significantly increased after treatment with PDGF-BB in a dose- and time-dependent manner *in vitro*, while the lentivirus knockdown BOP1 significantly inhibited the cell proliferation and migration *in vitro*, and potently alleviated the neointimal hyperplasia *in vivo*. Furthermore, BOP1 was highly expressed and colocalized with Ki-67 in cells on the inside edge of the neointima, compared with the media of low proliferation and migration ability, which suggests that BOP1 may be a crucial regulator in driving VSMC proliferation and migration. Hence, BOP1 may become an effective and adjustable molecular target for neointimal hyperplasia.

As the core component of the PeBoW complex, BOP1 is mainly involved in the process of pre-rRNA maturation and ribosome biogenesis. Ribosome biogenesis consumes approximately 80% energy of proliferative cells [32]. Recent studies have reported that the microRNA-712 derived from pre-rRNA have been proven to accelerate the atherosclerosis by inducing endothelial inflammation [39]. All these above implicate that the PeBoW complex may deeply be involved in the occurrence of neointimal hyperplasia. Unlike the other two components, WDR12 and PES1, BOP1 owns a PEST motif, which is a common peptide motif in most short-lived proteins [8]. This allows cells to timely respond to the excessive stimuli [40]. A previous study confirmed that the assembly and integrity of the PeBoW complex are highly sensitive to changes in BOP1 protein levels, rather than WDR12 and PES1 [8]. In addition, the effects of PES1 on both cell proliferation and ribosome synthesis are dependent on the interaction with BOP1 [41]. Nevertheless, BOP1 is necessary for the nucleolar accumulation of PES1. Therefore, we attempted to manipulate and validate its vital role in the neointimal hyperplasia, rather than WDR12 and PES1.

The rRNA and r-protein assemble into ribosomes and participate in a number of physiological actions [42]. Knockdown of BOP1, rather than complete knockout or replace with functional mutants, affected the cellular biological function but does not significantly impair the cellular structure and affect the survival, as previously confirmed [10, 12]. The inhibited expression of BOP1 suppressed rRNA maturation and subsequently resulted in the accumulation of free r-proteins, which may induce nucleolar stress. However, it is likely that the expression and role of r-proteins in different vascular cell lines would be quite different. Early studies have reported that RPL31 is suppressed in proliferative VSMCs [43], while the deficiency of RPL31 in macrophages would accelerate atherosclerosis by suppressing the synthesis of a series inflammatory proteins [44]. The present research revealed that there was no significant change in expression levels, but the nucleolar accumulation of RPL11 was suppressed after BOP1 knockdown in VSMCs, while RPL11 siRNA partly reserved the biological effects induced by LV-BOP1. In addition, the synthesis rate of nascent proteins slowed down after BOP1 knockdown. Hence, BOP1 may interfere with the neointimal hyperplasia via ribosome biogenesis and the nucleoplasm accumulation of free ribosome proteins.

Nucleolar stress is caused by the impaired ribosome biogenesis. In this case, the p53-dependent nucleolar stress signaling pathway may activate via the inhibition of MDM2 through free ribosomal proteins [45, 46], which were notably, RPL11 and RPL5 [35, 47, 48]. RPL11, as a component of 5S RNP, and the central role of the p53-dependent nucleolar stress signaling pathway have been proven to play the most significant role in MDM2 inhibition by directly binding the zinc finger of MDM2 to the hydrophilic residues [49]. The accumulation of RPL11 in the nucleoplasm and the combination with MDM2 may be the crucial mechanism of both p53 activation and MDM2 inhibition [28]. The present data shows that RPL11 and MDM2 colocalized in the nucleoplasm in VSMCs after BOP1 knockdown and that the binding of endogenous MDM2 to RPL11 was much greater than that of VSMCs transfected with LV-SCR.

A number of evidences have indicated that the p53-MDM2 signal pathway plays a vital role in pathogenesis of neointimal hyperplasia. Data from several studies suggested that inhibition of MDM2 attenuates neointimal hyperplasia via suppressing the vascular proliferation and inflammation [50] and that LincRNA-p21 also regulates neointima hyperplasia by enhancing the p53 activity [51]. The present result revealed that p53 significantly upregulated in protein levels, and the area of neointima obviously decreased in the carotid of mice after BOP1 knockdown by the lentivirus. In addition, all the above effects can be mostly eliminated in p53^−/−^ mice, consist with p53 inhibition by PFT-*α* in HVSMCs, confirms that p53-MDM2 as the downstream element of BOP1 in neointimal hyperplasia.

*In vitro*, our study further showed that BOP1 knockdown blocked cell cycle of HVSMCs in G0/G1 stage, which could be partly reserved by RPL11 siRNA or inhibiting p53. As a major mediator of cell cycle G1 arrest induced by p53, p21 protein levels increased after BOP1 knockdown and decreased with p53 inhibition, consistent with previous researches [52, 53]. As the two most obvious biological processes of p53 activation, cell cycle is closely related to apoptosis [54]. A large number of studies have confirmed that p53 targets involved in cell cycle and DNA repair often have a higher affinity binding sites, whereas high level p53 under the continuous stimulation of injury factors will simultaneously activate the connection sites of apoptosis-related genes, consistent with this, upregulated HVSMC apoptosis rate after BOP1 knockdown was observed [55, 56]. Besides, BOP1 knockdown also increased the level of intracellular ROS, which was further evidenced by the upregulation of 8-OHdG expression. Numerous studies have shown that there is a complex dynamic regulatory network between ROS and p53, except for the important factors of intermodulation; the balance of each is also affected by multiple other factors [57, 58]. Although we cannot determine whether BOP1 knockdown directly regulates ROS production or induces the increased ROS level in a p53-dependent manner and subsequently facilitates ROS-triggered apoptosis, both ultimately promote the inhibition of neointimal hyperplasia. As the mainstream studies believe that p53 activation is the protective effect of cells stimulated by ribosomal dysfunction induced by nucleolar stress, and the RPL11 knockout data in the current study, it seems that upregulation of p53 cannot be simply reversed by antioxidants, although more rigorous experiments are still needed to confirm this.

Besides, PFT-*α* has recently been reported to decrease the level of intracellular ROS in MCF7 cells through activation of an aryl hydrocarbon receptor- (AHR-) Nrf2 axis in a p53-independent manner [59]. This undoubtedly compromised the validity of using PFT-*α* to confirm the association between p53 and ROS and may have important guiding significance for the future use of PFT-*α* on p53 inhibition. However, it has been also found in other studies that PFT-*α* enhanced Nrf2 gene expression and decreased ROS levels through a p53-dependent manner in the kidney cortex [60], and no definitive studies has demonstrated that PFT-*α* reduces ROS in VSMCs in a p53-independent manner. Therefore, further studies about antioxidant properties of PFT-*α* in different cell lines are still needed.

Our previous study revealed that the BOP1 knockdown may induce AMD through the upregulation of p53 [13]. As BOP1 is highly conserved in mammalian cells, and ribosome biogenesis also plays critical functions within normal cells, we tested the local intervention approach to avoid system side effects, in line with the practice of the clinical stent system. Just like we thought, no carotid aneurysms or deaths were observed in the current study. Besides, the occurrence of aneurysms is closely related to hemodynamics [61], while the coronary arteries are less affected by hemodynamics changes and thus may make the local intervention safer.

One limitation of the present study was that the SMC-specific knockout or knockdown in animals was not available for the study and that BOP1 is highly conserved in VSMCs, as well as in other vascular cells, such as endothelial cells and macrophages. Although VSMCs are essential for neointimal hyperplasia, the specific intervention of BOP1 in VSMCs would make the present study more convincing. Another limitation is that there was an emerging field of evidence suggesting that the existence of a number of alternative nucleolar stress signaling pathways that involved non-r-proteins [16] and a number of alternative nucleolar stress pathways that involved nucleolar factors that bypass p53 directly play crucial roles in apoptosis, which are known as p53-independent stress signaling pathways [14]. The present research group will continue to conduct additional research for these issues.

In conclusion, the present study depicted that BOP1 knockdown significantly inhibited VSMC proliferation and migration *in vitro*, and subsequently, the neointimal hyperplasia *in vivo* via both the RPL11/MDM2/p53 nucleolar stress response pathway and nascent protein synthesis inhibition. Importantly, BOP1 is highly located on the inside edge of neointima, which makes BOP1 a promising target that may easily be interfered by drug-covered stent through intervention.

## Data Availability

The data used to support the findings of this study are available from the corresponding author upon request.

## Supplementary Materials

Supplementary Materials Supplementary Table 1: clinical characters of the patients enrolled in this study. Supplementary Figure S1: BOP1 knockdown decreased the rate of nascent protein synthesis and cannot be restored by PFT-*α*. (A) HVSMCs were transfected with LV-BOP1 or LV-SCR combined with 10 *μ*M PFT-*α* or DMSO treatment for 48 hours and administrated with puromycin (1 *μ*g/ml) for 40 minutes to label the nascent proteins (antipuromycin). The total protein was detected by Coomassie blue staining after SDS-PAGE electrophoresis (Coomassie blue). (B) The statistical analysis of the ratio of nascent proteins to the total protein is shown (*n* = 3).
