# Supplementary material for: BOP1 Knockdown Attenuates Neointimal Hyperplasia by Activating p53 and Inhibiting Nascent Protein Synthesis
Source: Oxid Med Cell Longev. 2021 Jan 16;2021:5986260. doi: 10.1155/2021/5986260 (PMC7826231; doi:10.1155/2021/5986260)
Supplement: Supplementary Materials — Supplementary Table 1: clinical characters of the patients enrolled in this study. Supplementary Figure S1: BOP1 knockdown decreased the rate of nascent protein synthesis and cannot be restored by PFT-α. (A) HVSMCs were transfected with LV-BOP1 or LV-SCR combined with 10 μM PFT-α or DMSO treatment for 48 hours and administrated with puromycin (1 μg/ml) for 40 minutes to label the nascent proteins (antipuromycin). The total protein was detected by Coomassie blue staining after SDS-PAGE electrophoresis (Coomassie blue). (B) The statistical analysis of the ratio of nascent proteins to the total protein is shown (n = 3). [file 5986260.f1.docx]

**SUPPLEMENTARY MATERIAL**

Supplementary Table 1: Clinical characters of the patients enrolled in this study.

|  | AS(n=9) | PCI(n=9) | Normal(n=9) | P value |
| --- | --- | --- | --- | --- |
| Sex,males(%) | 77.8 | 77.8 | 66.7 | 0.8246& |
| Age,y(mean±SD) | 58.7±7.6 | 53.9±8.6 | 55.8±3.6 | 0.3938# |
| Hypertension(%) | 55.6 | 33.3 | 33.3 | 0.5414& |
| Diebetes mellitus(%) | 22.2 | 22.2 | 11.1 | 0.7823& |
| Dyslipidemia(%) | 77.8 | 66.7 | 44.4 | 0.3290& |
| Previous MI(%) | 77.8 | 88.9 | 0.0 | 0.0002*& |

#,One-Way ANOVA test; &,Fisher's test; *,P＜0.05

**Supplementary Figure S1**


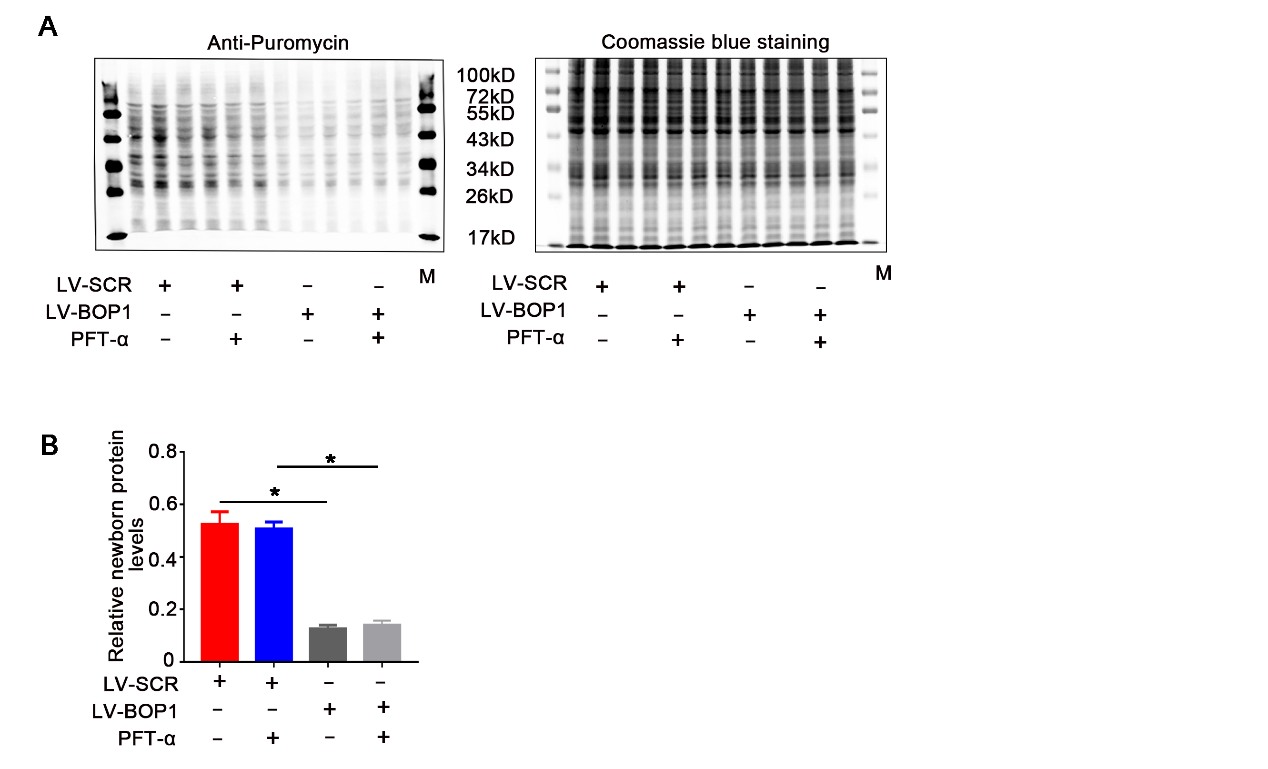


**Supplementary Figure S1 BOP1 knockdown decreased the rate of nascent protein synthesis and can’t be restored by PFT-α.**

(A) HVSMCs were transfected with LV-BOP1 or LV-SCR combined with 10μM PFT-α or DMSO treatment for 48 hours, and administrated with puromycin (1 μg/ml) for 40 minutes to label the nascent proteins (Anti-puromycin). The total protein was detected by Coomassie blue staining after SDS-PAGE electrophoresis (Coomassie blue). (B) The statistical analysis of the ratio of nascent proteins to the total protein is shown (n=3).
